# Supplementary material for: Genome-Wide Identification and Expression Analysis Reveals Roles of the NRAMP Gene Family in Iron/Cadmium Interactions in Peanut
Source: Int J Mol Sci. 2023 Jan 15;24(2):1713. doi: 10.3390/ijms24021713 (PMC9866697; doi:10.3390/ijms24021713)
Supplement: Supplementary file 1 [file ijms-24-01713-s001.zip › supplementary figures.pdf]

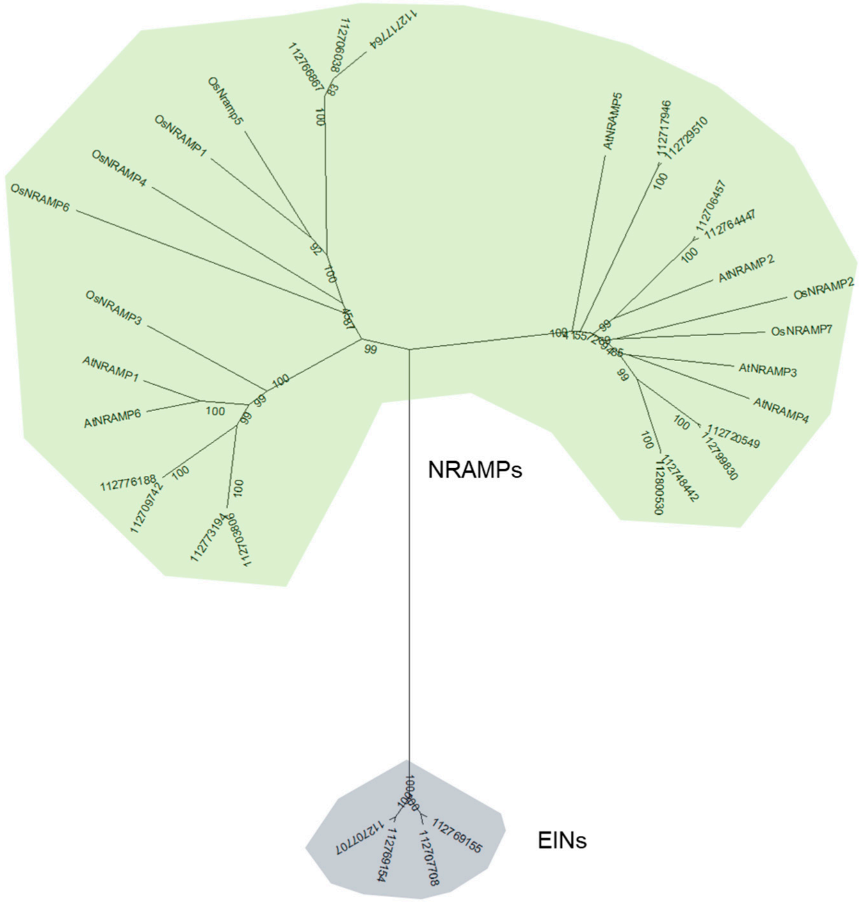

Figure S1. Screening NRAMPs from candidate sequences identified by a BLASTP search against the peanut genome using phylogenetic analysis with AtNRAMPs from *Arabidopsis* and OsNRAMPs from rice. A total of 15 protein sequences were identified to be putative AhNRAMP members, which were named based on phylogenetic relationships with AtNRAMPs from *Arabidopsis*.

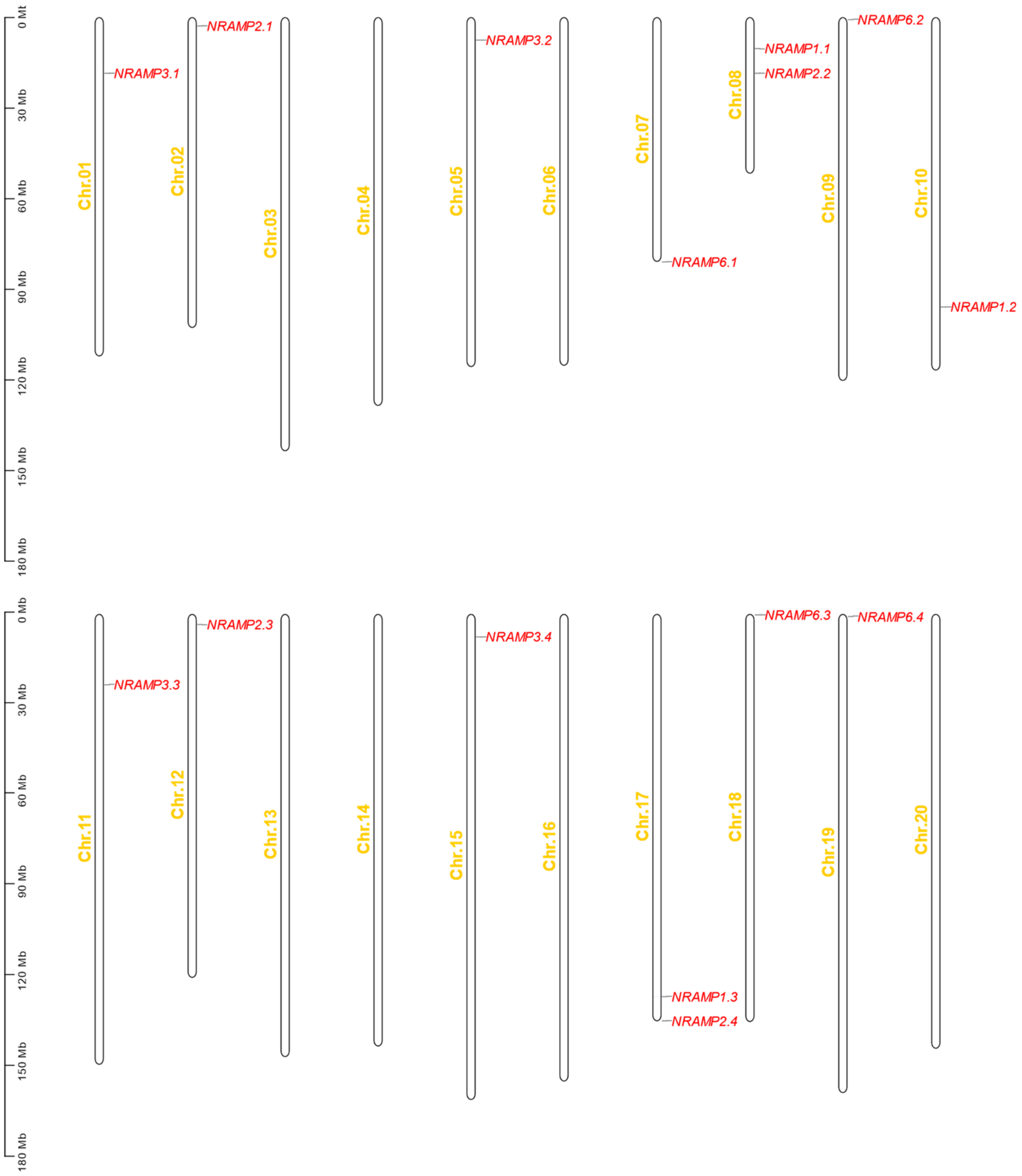

Figure S2. Distribution of *AhNRAMP* genes on the chromosomes of peanut. Chromosome numbers are shown at the left of the bars. *AhNRAMP* genes are labeled at the right of the chromosomes. Scale bar on the left indicates the chromosome lengths (Mb).

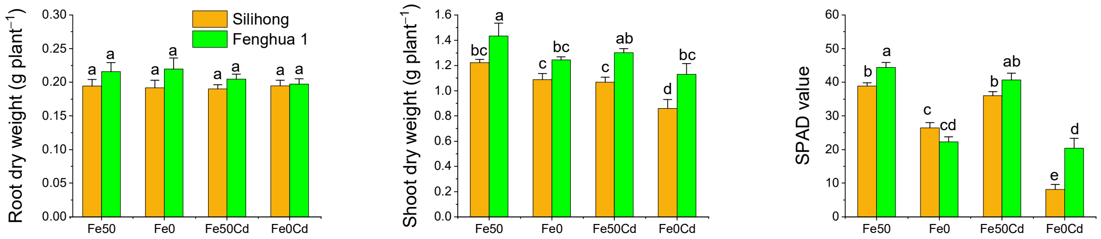

Figure S3. Biomass accumulation and leaf chlorophyll content (SPAD value) of two peanut cultivars exposed to 0 or 2 μM Cd under Fe-sufficient (Fe50) and Fe-deficient (Fe0) conditions for 14 days. Data (means ± SE, n = 3 for dry weight and 6 for SPAD value) sharing the same letter(s) above the error bars are not significantly different at the 0.05 level based on the Duncan multiple range test.
